# Thalidomide and dexamethasone vs. bortezomib and dexamethasone for melphalan refractory myeloma: a randomized study

**DOI:** 10.1111/j.1600-0609.2012.01775.x

**Published:** 2012-06

**Authors:** Martin Hjorth, Øyvind Hjertner, Lene Meldgaard Knudsen, Nina Gulbrandsen, Erik Holmberg, Per Trøllund Pedersen, Niels Frost Andersen, Björn Andréasson, Rolf Billström, Kristina Carlson, Margaretha S Carlsson, Max Flogegård, Karin Forsberg, Peter Gimsing, Torbjörn Karlsson, Olle Linder, Hareth Nahi, Annika Othzén, Agneta Swedin

**Affiliations:** 1 Department of Medicine, Lidköping HospitalLidköping, Sweden; 2 Department of Hematology, St Olavs University Hospital, and Department of Cancer Research and Molecular Medicine, Norwegian University of Science and TechnologyTrondheim, Norway; 3 Department of Hematology, Odense University HospitalOdense, Denmark; 4 Department of Hematology and Faculty Division, Ullevål University HospitalOslo, Norway; 5 Department of Oncology, Institute of Clinical Sciences, Sahlgrenska Academy at University of GothenburgGothenburg, Sweden; 6 Department of Hematology, Esbjerg HospitalEsbjerg; 7 Department of Hematology, Aarhus University HospitalAarhus, Denmark; 8 Hematology Section, Department of Medicine, NU Health OrganizationUddevalla; 9 Department of Medicine, Skövde Hospital/KSSSkövde; 10 Department of Hematology, Uppsala University HospitalUppsala, Sweden; 11 Department of Medicine, Växjö HospitalVäxjö; 12 Department of Medicine, Falun HospitalFalun; 13 Department of Hematology, Norrland University HospitalUmeå, Sweden; 14 Department of Hematology, Rigshospitalet and University of CopenhagenCopenhagen, Denmark; 15 Department of Hematology, Örebro University HospitalÖrebro; 16 Division of Hematology, Department of Medicine, Karolinska InstitutetHuddinge, Stockholm; 17 Department of Medicine, Gävle HospitalGävle; 18 Department of Hematology, Skane University HospitalLund, Sweden

**Keywords:** multiple myeloma, thalidomide, bortezomib, randomized trial, quality of life

## Abstract

**Objectives:**

Thalidomide and bortezomib have been frequently used for second-line therapy in patients with myeloma relapsing after or refractory to initial melphalan-based treatment, but no randomized trials have been published comparing these two treatment alternatives.

**Methods:**

Thalidomide- and bortezomib-naïve patients with melphalan refractory myeloma were randomly assigned to low-dose thalidomide + dexamethasone (Thal-Dex) or bortezomib + dexamethasone (Bort-Dex). At progression on either therapy, the patients were offered crossover to the alternative drug combination. An estimated 300 patients would be needed for the trial to detect a 50% difference in median PFS between the treatment arms.

**Results:**

After inclusion of 131 patients, the trial was prematurely closed because of low accrual. Sixty-seven patients were randomized to Thal-Dex and 64 to Bort-Dex. Progression-free survival was similar (median, 9.0 months for Thal-Dex and 7.2 for Bort-Dex). Response rate was similar (55% for Thal-Dex and 63% for Bort-Dex), but time to response was shorter (*P* < 0.05) and the VGPR rate higher (*P* < 0.01) for Bort-Dex. Time-to-other treatment after crossover was similar (median, 13.2 months for Thal-Dex and 11.2 months for Bort-Dex), as was overall survival (22.8 months for Thal-Dex and 19.0 for Bort-Dex). Venous thromboembolism was seen in seven patients and cerebrovascular events in four patients in the Thal-Dex group. Severe neuropathy, reactivation of herpes virus infections, and mental depression were more frequently observed in the Bort-Dex group. In the quality-of-life analysis, no difference was noted for physical function, pain, and global quality of life. Fatigue and sleep disturbances were significantly more prevalent in the Bort-Dex group.

**Conclusions:**

Thalidomide (50–100 mg daily) in combination with dexamethasone seems to have an efficacy comparable with that of bortezomib and dexamethasone in melphalan refractory myeloma. However, the statistical strength of the results in this study is limited by the low number of included patients.

The treatment options for multiple myeloma have improved considerably in recent years, both as concerns initial therapy and treatment for refractory and relapsing disease. In 2007, when this study was initiated, there was still a consensus in most centers on the use of high-dose melphalan with autologous stem cell support for initial therapy in younger patients and melphalan–prednisone in elderly patients (1). For relapsed and refractory disease, however, few randomized clinical trials had been performed and no consensus on best choice of therapy was present. Thalidomide was frequently used since the initial report on favorable results in 1999 (2). A systematic review of published phase II studies on thalidomide monotherapy reported a 29% response rate (3). The addition of dexamethasone to thalidomide was reported to increase the response rate to 46% with a median event-free survival of 8 months (4). However, no randomized trials were carried out, and thalidomide was not approved by the European authorities. Bortezomib was introduced in 2006 and approved after the publications of two phase II studies (5, 6) and one phase III study comparing bortezomib with high-dose dexamethasone (7, 8). Briefly, these studies reported a response rate of 30–43% for bortezomib monotherapy, increasing 10–15% after the addition of dexamethasone, and a median time to progression of 7 months. The phase III trial (7) showed a superiority of bortezomib over dexamethasone. In the setting of this study, no randomized studies have been published comparing thalidomide with bortezomib, nor any studies comparing any of these drugs with other chemotherapy.

The purpose of this study was to compare thalidomide + dexamethasone with bortezomib + dexamethasone for efficacy, toxicity, and quality of life (QoL) in patients with melphalan refractory myeloma. Progression-free survival (PFS) was the primary endpoint. Secondary endpoints were response rate and duration, toxicity, QoL, time to next treatment, and overall survival. The study was carried out in a multicenter setting involving several local hospitals. In our study, we used drug doses considered the current best standard treatment, which reflects day-to-day clinical practice. This was a genuinely academic study without any support or involvement from the pharmaceutical industry.

Since 2008, when the trial was opened for patient inclusions, the prerequisites for the study have changed. Both thalidomide and bortezomib have been approved for use in combination with melphalan and prednisone for initial therapy and lenalidomide for relapsed and refractory myeloma. For these reasons, the accrual rate of the study dropped and the study was prematurely closed before half of the projected number of patients was included. Nevertheless, no other randomized trial comparing thalidomide with bortezomib has been published, and the results of the trial are still of relevance for patients who have not received thalidomide or bortezomib as part of initial therapy, as well as for patients relapsing after high-dose melphalan.

The study was registered at ClinicalTrials.gov. identifier: NCT00602511.

## Design and methods

### Patients

This open phase III randomized multicenter trial was conducted in 29 hospitals in Sweden, Denmark, and Norway. From October 2007 to September 2010, 131 patients entered the study (60 were recruited from university hospitals and 71 from local hospitals). Eligible for the study was patients of any age who had treatment demanding myeloma and were refractory to melphalan defined by (i) the absence of response or progression on initial melphalan-based treatment (primary refractory disease), (ii) the absence of response or progression on reinstituted melphalan treatment after previous response (relapsed and refractory), and (iii) treatment demanding relapse after previous response if occurring within 12 months after high-dose therapy or within 6 months after oral melphalan treatment (relapsing disease). However, even patients with later relapse could be included if further melphalan-based therapy was regarded as futile according to the responsible physician. Exclusion criteria were (i) former treatment with thalidomide, bortezomib, or lenalidomide; (ii) sensory neuropathy (grade 3 or more) or neuropathic pain (grade 2 or more); (iii) platelet counts <25 × 10^9^/L; (iv) severe comorbidity; (v) transformation to plasma cell leukemia or aggressive lymphoma; and (vi) non-secreting myeloma without abnormal free light chain ratio. The study was conducted in accordance with the Helsinki declaration and was approved by ethical committees in Sweden, Denmark, and Norway.

### Statistical considerations

We estimated that 300 patients would be needed for the trial assuming a median PFS in the control arm of 7 months, a 50% difference in median PFS between the treatment arms, an accrual rate of 12 patients per month during 25 months and a 4-month follow-up after the last included patient, and a PFS analysis with a statistical power of 80% and a significance level of 5%.

### Study design

*Randomization:* After stratification for previously given high-dose melphalan or not, patients were randomized 1 : 1 to receive treatment with either thalidomide and dexamethasone (Thal-Dex) or bortezomib and dexamethasone (Bort-Dex). The randomization procedure was centralized and performed electronically by a web-based form.

*Treatment according to randomization:* In the Thal-Dex group, thalidomide was given at a dose of 50 mg once daily initially, escalated by 50 mg every 3 wk to a maximum of 200 mg daily, unless sufficient response was achieved by a lower dose. The dexamethasone dose was 40 mg on days 1–4, repeated every third week. In the Bort-Dex group, bortezomib was given with 1.3 mg/m^2^ intravenously on days 1, 4, 8, and 11 of a 3-wk cycle. The dexamethasone dose was 20 mg on days 1–2, 4–5, 8–9, and 11–12. In both groups, treatment was continued until the achievement of best response followed by 1–2 additional 3-wk treatment cycles, followed by a treatment pause. In case of progression during the treatment pause, the initial treatment was reinstituted and continued until definitive failure. In case of neurotoxicity or other dose-dependent side effects, the doses of thalidomide and bortezomib were reduced. For bortezomib neurotoxicity, internationally accepted guidelines were followed. The initial dose of dexamethasone was given for two treatment cycles and thereafter individualized depending on response and side effects.

*Crossover treatment:* Patients with definitive failure in the Thal-Dex group were offered crossover treatment with bortezomib and dexamethasone. Similarly, patients with failure in the Bort-Dex group received crossover treatment with thalidomide and dexamethasone. The principles, dosing, schedules, and duration of treatment were the same after crossover as for initial treatment according to randomization.

*Concomitant medication:* Antithrombotic prophylaxis and acyclovir prophylaxis were not mandatory according to the study protocol but used routinely in an increasing proportion of participating centers during the study period.

*Follow-up:* Patients were followed every 3 wk until the achievement of response and plateau phase or at least four treatment cycles; thereafter, follow-up was every 3–6 wk until the patient reached the end of the study, defined by death or start of other treatment off-protocol. At each visit, a response evaluation was performed after laboratory check-up of relevant tests, including M protein in blood or urine or serum free light lambda chains, as well as toxicity evaluation according to common terminology criteria for adverse events (CTCAE v3.0) (9). All patients were followed until death or until January 2011.

*Quality-of-life assessment:* Health-related QoL (HRQoL) was measured by the European Organization of Research and Treatment of Cancer QLQ-C30 questionnaire. It is constructed to be cancer specific and multidimensional and has been validated for MM patients. The questionnaire incorporates five functional scales (physical, role, emotional, cognitive, and social functioning), three symptom scales (pain, fatigue, and nausea/vomiting), a global health and quality-of-life scale, and a number of single items (appetite loss, dyspnea, diarrhea, constipation, sleep disturbances, and financial impact). All scale and item scores were linearly transformed, so that the results ranged from 0 to 100. For the five functioning scales and the single global health/quality-of-life scale, higher scores represent higher level of functioning. For the three symptom scales and the single symptom items, higher scores represent higher level of symptoms. The patients completed the questionnaires before randomization and thereby before start of treatment, and later mailed to the patients after 6, 12 wk, 6 months, and thereafter every 6 months until the patient reached the end of the study.

### Definitions

Partial response (PR), very good partial response (VGPR), and progressive disease (PD) were defined according to the IMWG guidelines (10). Failure was defined by (i) PD during treatment, (ii) stable disease, that is, neither PR nor PD, but with remaining treatment demanding ROTI despite treatment with optimal drug doses, (iii) PD on reinstituted treatment after previous response, and (iv) PD during treatment pause in responding patients in which reinstituted treatment was contraindicated because of toxicity.

PFS was calculated from the time of randomization until definitive failure on treatment according to randomization, or death. Time to response was calculated from randomization until the achievement of at least PR and response duration from that time until definitive failure. Time to next treatment after the first treatment line was calculated from randomization until start of crossover treatment, other treatment off-protocol, or death, whichever came first. Time to other treatment off-protocol was calculated from randomization until start of other treatment off-protocol, or death. Overall survival was calculated from the time of randomization.

### Statistical methods

Comparisons between groups for PFS, time to response, response duration, time to other treatment, and overall survival were performed by a two-sided log-rank test based on Kaplan–Meier curves. Comparisons between groups for response rates were performed by Fisher's exact test. Cox's regression test was used to analyze prognostic factors. The principle of intention-to-treat was applied in all analyses, except time to response and response duration, in which only responding patients were included. The patients’ self-assessments of QoL using the QLQ-C30 were analyzed using the Mann–Whitney test for related samples. Differences or changes of 10 or more on a 0–100 scale were regarded as clinically significant (11). As a large number of comparisons were performed, in the QoL analysis, a *P*-value of <0.01 was considered necessary for statistical significance.

## Results

### Evaluable patients

**Figure 1:**
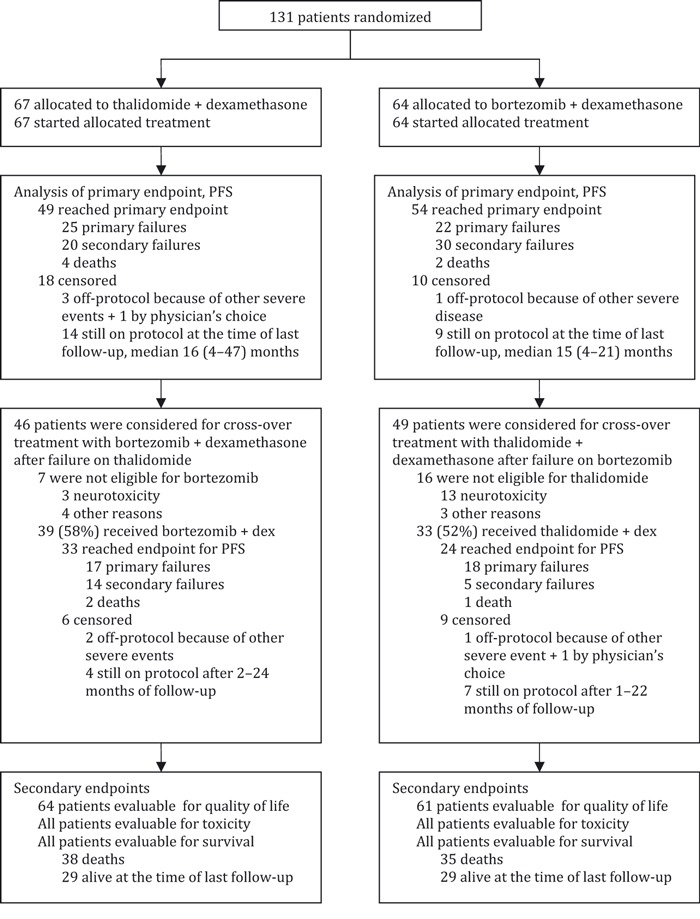
Flow diagram of randomized patients.

Sixty-seven patients were randomized to Thal-Dex and 64 to Bort-Dex (Fig. 1). All patients are included in the analysis of progression-free survival. All are also included in the analyses of response, toxicity, time to other treatment, and survival. All these analyses are performed according to the principle of intention-to-treat.

### Patient characteristics

Patients’ clinical and laboratory characteristics at the time of randomization are shown in Table 1. About half of the patients in both groups were previously treated with high-dose melphalan with autologous stem cell support. 30% of patients in the Thal-Dex group and 25% in the Bort-Dex group were true refractory to melphalan. The remaining patients had relapsed off-therapy after previous response or plateau phase. There were significantly more women in the Thal-Dex group. However, in a univariate analysis, no prognostic importance of gender for PFS was found. For all other patient characteristics, the treatment arms were well balanced.

**Table 1:** Patient characteristics at the time of randomization

	Thal-Dex (*n* = 67)	Bort-Dex (*n* = 64)
Age (years), Median (range)	71 (38–85)	71 (50–84)
Sex, Male/Female	28/39	41/23
Time from diagnosis to randomization (months), Median (range)	28 (2–426)	29 (5–122)
Treated with high-dose melphalan, Number of patients (%)	33 (49)	33 (52)
Time from high-dose melphalan to randomization (months), Median (range)	12 (1–48)	12 (1–68)
High-dose melphalan not given, Number of patients (%)	34 (51)	31 (48)
Time from last melphalan dose to randomization (months), Median (range)	3 (1–28)	4 (1–26)
Patient category^1^	No. of patients	No. of patients
Refractory disease		
Primary refractory	11	10
Relapsed and refractory	9	6
Relapsing disease		
Smoldering relapse	13	14
Classical form of relapse	32	34
Plasmacytoma form	2	0
Performance status (WHO)		
0–1	53	52
2–3	14	12
M-protein class		
IgG	47	35
IgA	12	20
IgD	0	2
Light chains only	8	7
Plasma creatinine		
>200 μmol/L	2	0
Blood hemoglobin		
<100 g/L	22	23
Plasma calcium		
>2.6 mmol/L or ionized calcium >1.3 mmol/L	8	3
Plasma albumin		
≤35 g/L	36	30
Serum β-2-microglobulin		
<3.5 mg/L	18	21
3.5–5.5 mg/L	18	24
>5.5 mg/L	20	12
Missing data	11	7
Skeletal lesions on X-ray		
None	11	12
Present	56	51
Sensory neuropathy (WHO grade)		
0	48	49
1	18	12
2	1	3
Neuropathic pain (WHO grade)		
0	60	60
1	7	4

1 Patient categories: Refractory disease - no response to melphalan or progression during ongoing melphalan treatment, either during initial therapy (Primary refractory disease) or during re-instituted therapy after response (Relapsed and refractory). Insidious relapse - increase in M-protein concentration in serum or urine (biochemical relapse) during more than 100 days before clinical signs of relapse. Classical form of relapse - clinical signs of relapse within 100 days after biochemically defined relapse. Plasmacytoma form of relapse - skeletal or extraskeletal plasmacytoma with minor or no other signs of relapse.

### Dose intensity

*Thalidomide:* The initial dose of thalidomide was 50 mg/d in 66 patients and 100 mg/d in 1 patient in the Thal-Dex group. The dose was escalated to a maximum of 100 mg in 26 patients (39%), 150 mg in 6 (9%), and 200 mg in 5 (7%). The median duration of thalidomide treatment was 5.1 months.

*Bortezomib:* All patients in the Bort-Dex group received at least one full cycle of bortezomib. The median number of completed cycles was four; and the median time on bortezomib treatment was 3.5 months. In 19 patients (30%), the dose of bortezomib was reduced because of toxicity in the second or later treatment cycle. The remaining 45 patients received full dose until treatment was stopped because of response, progression, or toxicity.

*Dexamethasone:* The dose of dexamethasone given during the first 3-wk treatment cycle was 160 mg in 65 patients (97%) in the Thal-Dex group and 58 patients (91%) in the Bort-Dex group. Remaining patients received 80 mg per 3-wk cycle initially. The median time on dexamethasone treatment was 4.0 months for Thal-Dex and 3.5 months for Bort-Dex, and the median total given dose of dexamethasone was 640 and 560 mg for Thal-Dex and Bort-Dex, respectively.

### Concomitant medication

Twenty-one of 67 patients (31%) in the Thal-Dex group and five of 64 (10%) in the Bort-Dex group received some form of anticoagulant treatment, usually aspirin 75 mg daily. Acyclovir was given in nine patients (13%) in the Thal-Dex group and in 28 (44%) in the Bort-Dex group trimethoprim–sulfamethoxazole was given to six patients (9%) in the Thal-Dex group and to 10 (16%) in the Bort-Dex group; and erythropoietin was given to 10 patients (15%) in the Thal-Dex group and to six patients (9%) in the Bort-Dex group.

### Response evaluation

At least PR was achieved in 55% of the patients treated with Thal-Dex and in 63% of the patients treated with Bort-Dex, a difference that did not reach statistical significance (Table 2). However, the proportion of patients reaching VGPR was significantly higher in the Bort-Dex group: 36% vs. 13% (*P* < 0.01). No statistically significant difference was demonstrated between patients previously treated with high-dose melphalan and those not; nor was there a significant difference between refractory and relapsing patients.

**Table 2:** Response rate

Treatment according to randomization	Thal-Dex (*n* = 67)	Bort-Dex (*n* = 64)	
PR + VGPR	37 (55%)	40 (63%)	n.s.
VGPR	9 (13%)	23 (36%)	*P* < 0.01
No response	30 (45%)	24 (38%)	
Crossover treatment after failure	Bortezomib + Dex (*n* = 39)	Thalidomide + Dex (*n* = 33)	
PR + VGPR	18 (46%)	10 (30%)	
VGPR	8 (21%)	4 (12%)	
No response	21 (54%)	23 (70%)	

For responding patients, the time to response from start of therapy was shorter for Bort-Dex, with a median of 1.6 months (95% CI 1.4;2.3) vs. 3.0 months (95% CI 2.1;5.6) for Thal-Dex (*P* < 0.05). The response duration was similar in the two groups, with a median time of 9.9 months (95% CI 5.7;23.2) for Thal-Dex and 12.7 months (95% CI 5.4;15.3) for Bort-Dex. Further, the time to start of next line of treatment (all patients included) was similar for both groups, with a median of 9.7 months (95% CI 5.3;11.4) for Thal-Dex and 8.5 months (95% CI 4.5;11.8) for Bort-Dex.

### Progression-free survival

**Figure 2:**
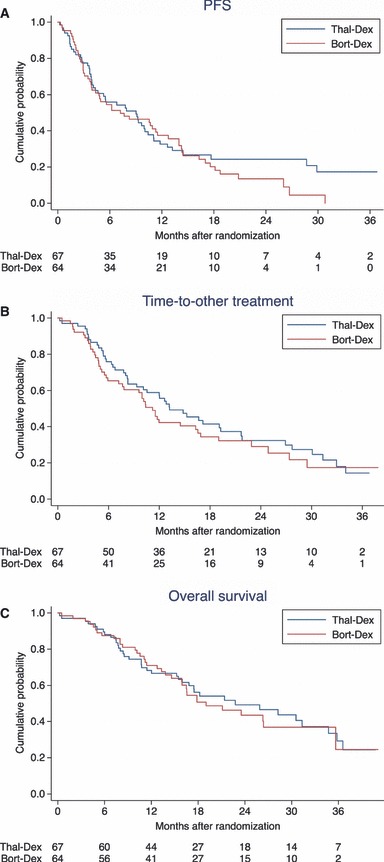
(A) Progression-free survival. (B) Time-to-other treatment after completed crossover therapy. (C) Overall survival from the time of randomization.

No difference in PFS was noted between the two patient groups (Fig. 2A). The median PFS time was 9.0 months (95% CI 4.3;10.4) for Thal-Dex and 7.2 months (95% CI 3.9;11.5) for Bort-Dex.

### Prognostic factors

In a univariate analysis, serum β-2-microglobulin, plasma albumin, and plasma creatinine were found to have prognostic importance for PFS. No prognostic importance was found for age, gender, previous high-dose therapy, refractoriness vs. relapse, WHO performance status, blood hemoglobin, plasma calcium, or M-protein class. In a multivariate analysis, however, only serum β-2-microglobulin was found to have independent prognostic importance for PFS (*P* < 0.001, hazard rate 1.10 (95% CI 1.05;1.17)).

### Crossover treatment

Thirty-nine patients received bortezomib + dexamethasone after failure on Thal-Dex, while 33 received thalidomide + dexamethasone after failure on Bort-Dex. The reasons for not giving crossover treatment are given in Fig. 1. The main reason was neuropathy precluding thalidomide therapy in 13 previously bortezomib-treated patients and bortezomib in three previously thalidomide-treated patients. In the Thal-Dex group, 18 patients (46%) reached an at least PR on crossover treatment with bortezomib + dexamethasone. In the Bort-Dex group, 10 patients (30%) responded to thalidomide + dexamethasone.

### Time-to-other treatment

The total effect of the combined thalidomide and bortezomib treatment was estimated by the time from randomization to start of the treatment off-protocol (Fig. 2B). Again, no statistically significant difference was found (*P* = 0.35). The median time to start of other treatment was 13.2 months (95% CI 9.3;19.3) in the Thal-Dex group and 11.2 months (95% CI 7.7;16.6) in the Bort-Dex group.

### Survival

Overall survival was similar in the two treatment groups (Fig. 2C). The median survival time from randomization was 22.8 months (95% CI 16.0;34.7) in the Thal-Dex group and 19.0 months (95% CI 15.9;35.6) in the Bort-Dex group.

### Toxicity

Toxicity during treatment according to randomization, that is, before crossover, is shown in Table 3.

**Table 3:** Toxicity The highest noted toxicity grade for each patient during treatment according to randomization

	Thal-Dex *n* = 67				Bort-Dex *n* = 64			
CTCAE grade	2	3	4	5	2	3	4	5
Sensory neuropathy	12	5			15	12		
Motor neuropathy	5	4			7	5		
Neuropathic pain	5				12	9		
Neutropenia	13	8	1		8	8	3	
Thrombocytopenia	7	3	1		11	19	3	
Hemorrhage		1			3	1		
Infections (Herpes excluded)	17	12	3	1	9	17	3	1
Herpes zoster or simplex	1	1			4	4		
Fever without documented infection	1				3	2		
Nausea and vomiting	3	1			1	2		
Diarrhea		1			6	1		
Constipation	14	5			11	3		
Somnolence	6	1			5	1		
Psychiatric reactions	10	1	1		6	5		
Vertigo	7	5			7	7		
Skin reactions	8				5			
Deep vein thrombosis and/or pulmonary embolism		5	2			1		
Cardiac failure	4	1	1		3	3		
Cerebrovascular events			3	1				
Pulmonary toxicity						2	1	
Insulin-dependent diabetes mellitus		3						
Other toxicity	12	2			13			

*Neurotoxicity* was observed more frequently in the Bort-Dex group than in the Thal-Dex group. Sensory or motor neuropathy (grade 3–4) was noted in 12 compared with six patients, and neuropathic pain (grade 2–4) was seen in 21 compared with 5.

*Infections* that are clinically or microbiologically documented (grade 3–5), herpes infections excluded, were detected in 16 patients in the Thal-Dex group and in 21 patients in the Bort-Dex group. About half of the cases in both groups were pneumonia. Reactivation of herpes zoster (grade 2–3) was seen in five patients in the Bort-Dex group and in one patient in the Thal-Dex group, while herpes simplex (grade 3) was found in two patients in the Bort-Dex group and in one patient in the Thal-Dex group. Only one of these patients, assigned to the Bort-Dex group, was on prophylactic acyclovir treatment at the time of the outbreak.

*Psychiatric reactions* (grade 3–4) were discovered in two patients in the Thal-Dex group, one of whom developed visual hallucinations and one severe anxiety. Five patients in the Bort-Dex group developed depressive reactions (grade 3–4).

*Deep vein thrombosis or pulmonary embolism* was observed in seven patients in the Thal-Dex group and in one patient in the Bort-Dex group. None of these patients was on prophylactic anticoagulant treatment at the time of the event.

Four severe *cerebrovascular events* occurred in the Thal-Dex group vs. none in the Bort-Dex group. One patient suffered a hemorrhagic stroke with a fatal outcome; one patient had a major ischemic stroke with subsequent sequel; and two patients had minor strokes without sequel. As for the patients with venous thromboembolism, none of these patients was on prophylactic anticoagulant treatment at the time of the event.

*Pulmonary and cardiac toxicity:* One patient developed acute pulmonary edema (grade 4) the day after the second injection of bortezomib. The cardiac function was found to be normal, and a bortezomib-induced pulmonary edema could not be excluded. Cardiac failure (grade 3–4) was seen in two patients in the Thal-Dex group and three in the Bort-Dex group. No case of myocardial infarction was noted.

*Toxicity during crossover treatment:* Toxicity after crossover was similar to that during treatment according to randomization. In the Thal-Dex group receiving bortezomib + dexamethasone after crossover, one patient had a minor ischemic stroke. In the Bort-Dex group receiving thalidomide + dexamethasone, one additional patient experienced deep vein thrombosis, one had a minor stroke, and one developed intestinal gangrene, probably from arterial obstruction, with a fatal outcome. None of these patients was on prophylactic anticoagulant treatment at the time of the event.

### Quality of life

**Figure 3:**
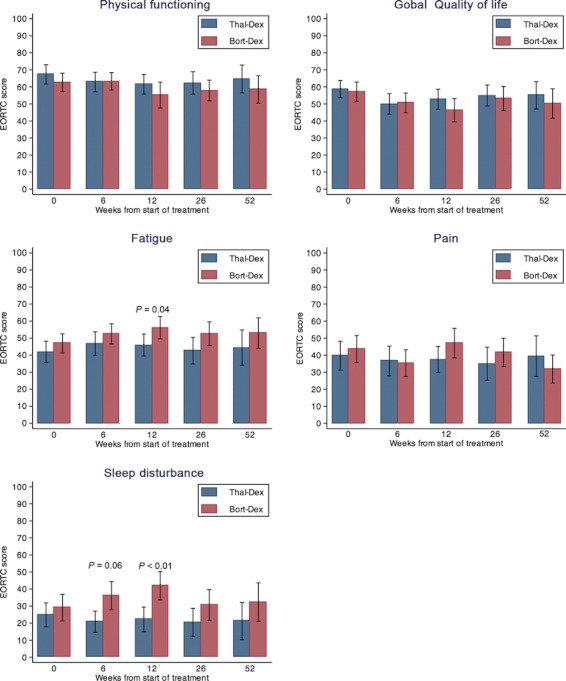
Self-reported QoL during treatment with Thal-Dex and Bort-Dex: mean scores at various times after the start of treatment. For physical functioning and global quality of life, higher scores indicate better functioning. For fatigue, pain, and sleep disturbances, higher scores indicate a higher level of symptoms.

The quality-of-life questionnaire (QLQ) was completed by 96% of patients still alive at 6 wk, 90% at 12 wk, and 76% at 6 months. The mean scores for the most important quality-of-life variables physical function, global quality of life, pain, and fatigue are shown in Fig. 3A–D. No improvement over time was seen for any of these variables. No differences were seen between the treatment groups beside fatigue, in which the scores for the Bort-Dex group was somewhat worse at 12 wk with a score difference of 10 (*P* = 0.04, ns).

Among other symptom-related QLQ variables, there was a higher score for sleep disturbances in the Bort-Dex group reaching statistical significance at 6 and 12 wk (Fig. 3E). The score increased by 14 points from the time of randomization and the difference amounted 20 points at 12 wk. For the remaining QoL variables, no significant differences were seen.

## Discussion

The choice of treatment for relapsing and refractory myeloma patients is challenging and hampered by the lack of randomized trials. Bortezomib and lenalidomide have both showed superiority to high-dose dexamethasone in relapse patients. However, high-dose dexamethasone is a treatment strategy that has never been regarded as standard treatment in the Nordic countries. Thus, in the absence of comparative trials for the practicing physician, it was not possible to make an evidence-based choice between thalidomide and bortezomib for melphalan refractory patients. This trial was designed to answer the question of whether any of these drugs was to be preferred before the other and, for patients in whom both drugs were planned to be used, whether there were any advantages to start with one or another.

The main result of this trial is that low-dose thalidomide (50–100 mg daily) in combination with dexamethasone has comparable efficacy as bortezomib + dexamethasone regarding PFS and time to next treatment for patients with melphalan refractory myeloma. Bortezomib was found to induce a more rapid response and a higher rate of VGPR, but this did not translate into a longer PFS or overall survival.

The median time on bortezomib (3.5 months) and thalidomide treatment (5.1 months) was short in this trial. The main reasons for early termination of treatment were therapeutic failure and toxicity. Peripheral neuropathy was a major concern, especially in the Bort-Dex group, and frequently the cause of early treatment discontinuation although we deliberately tried to limit this problem by early bortezomib dose reduction and a low starting dose for thalidomide.

The main strength of the study, additional to the unique design, was that it was performed in a multicenter setting with several local hospitals involved and that the doses of the study drugs were those generally used in the Nordic countries at the time of the trial, thereby reflecting everyday clinical practice.

The main weakness was that less than a half of the projected number of patients was included. The primary reason for this event was the introduction of thalidomide and bortezomib in the treatment strategies for initial therapy during the years 2007–2010, resulting in a drop in patient accrual and a premature closing of the study. However, the fact that response rates and PFS for both treatment arms are in the same magnitude as in other published trials (4–7) makes it probable that the results of this trial are reliable.

The time to other treatment off-protocol was similar for the two treatment groups, and we cannot answer the question of any probable advantage of giving thalidomide or bortezomib before the other. In this trial, there was a higher rate of neurotoxicity in the bortezomib group, thereby precluding crossover treatment with thalidomide. Recently, reports on single weekly bortezomib dosing and subcutaneous administration have shown a reduced rate of neurotoxicity without loss of efficacy (12, 13). Therefore, it cannot be excluded that the patients in the Bort-Dex group had done better with once weekly bortezomib instead of twice weekly that was the standard dose at the time of the study.

The spectrum of toxicity was mainly as expected. The limited number of patients in the study makes it difficult to compare the treatment arms for toxicity. Nevertheless, some observations are worth noting. There was a high rate of neurotoxicity in the Bort-Dex group, which underlines the necessity of a high grade of observance and dose reductions even at minor signs of neurotoxicity. A high but similar rate of severe infections was found in both treatment groups. Dexamethasone, which may have contributed to this event, was given in an equivalent total dose in the two treatment groups. The increased risk of herpes virus reactivations during bortezomib treatment has been noted in other studies (14) and underscores the recommendations of acyclovir prophylaxis (15). As concerns psychiatric toxicity, we observed several depressive reactions in the Bort-Dex group. However, the significance of this finding is not clear because of the low number of patients. This complication has not been described in other reports.

An important observation is the high number of vascular adverse events in the Thal-Dex group: seven patients experienced venous thromboembolism and four cerebrovascular complications. All these events occurred in those 46 patients who were not on thromboprophylactic medication. Three additional vascular events occurred during treatment with thalidomide and dexamethasone after cross-over. The risk of venous thromboembolism is well recognized since several years (16–18), whereas the risk of arterial obstruction has been observed more recently (19–21). The results of this study support the recommendation that anticoagulant prophylaxis should be given when the combination of thalidomide and dexamethasone is used for relapsed or refractory myeloma as well as for initial therapy (22, 23).

In contrast to what is ordinarily seen in the treatment of newly diagnosed myeloma (24, 25), the quality-of-life analysis did not reveal any improvement over time for either treatment group. The reasons for this could be that treatment in relapsing patients is initiated before the patient has as severe symptoms as in newly diagnosed disease and that the response rate is lower than for initial treatment. In the interpretation of QoL results, a score difference of 6–17 points or more is regarded as clinically important (11, 26). In this study, a difference of 10 was seen for fatigue, suggesting possible clinical importance albeit not reaching the statistical significance level of 0.01. For sleep disturbances, the score increment in the Bort-Dex group and the difference compared to the Thal-Dex group was both statistically significant and clinically important. The reason for the increase in sleep disturbances is not clear but may be related to neurotoxicity-dependent paresthesia and pains.

The value of prognostic factors is well established in newly diagnosed myeloma in which ISS stage and cytogenetics are used both for characterizing patients in clinical trials and for discussion of prognosis and choice of treatment for the individual patient (27, 28). In this trial, we found that serum β-2-microglobulin also had prognostic importance in the relapse setting. Therefore, β-2-microglobulin can also be used for prognostic purposes in relapsed and refractory patients.

In conclusion, low-dose thalidomide (50–100 mg daily), in combination with dexamethasone, seems to have an efficacy that is comparable with that of bortezomib and dexamethasone in melphalan refractory myeloma. In clinical practice, most patients will receive both drugs, one after the other. For the majority of patients, in the absence of differences in efficacy, the choice of initial treatment should be based on the differences in toxicity, QoL aspects, and other patient-related factors. For a patient with advanced disease with threatening complications, when a rapid response is desirable, bortezomib may be the preferred drug. For both drugs, measures should be taken to prevent evitable toxicity.

## Authorship

MH, **Ø**H, LMK, NG, and PTP designed the study, participated in patient enrollment and data acquisition and interpretation, and wrote the article. EH was responsible for data management and statistical analysis. NG was responsible for the QoL analysis. NFA, BA, RB, KC, MSC, MF, KF, PG, TK, OL, HN, AO, and AS participated in patient enrollment and data acquisition and reviewed the article. MH had the main responsibility for the study.

## Funding

This study was supported by grants from the Norwegian Research Council and by the Research Fund at Skaraborg Hospital, Sweden.

## Appendix

The following members and centers of the Nordic Myeloma Study Group participated in this study:

*Sweden*: Björn Andréasson, Uddevalla Hospital, Uddevalla; Martin Hjorth, Lidköping Hospital, Lidköping; Annika Othzén Jerner, Gävle Hospital, Gävle; Stig Lenhoff and Agneta Swedin, Lund University Hospital, Lund; Margaretha S Carlsson, Växjö Hospital, Växjö; Max Flogegård, Falun Hospital, Falun; Rolf Billström, Skövde Hospital/KSS, Skövde; Karin Forsberg, Norrland University Hospital, Umeå; Kristina Carlson, Uppsala University Hospital, Uppsala; Olle Linder, Örebro University Hospital, Örebro; Hareth Nahi, Karolinska University Hospital, Huddinge, Stockholm; Torbjörn Karlsson, Västerås Hospital, Västerås, and St. Göran Hospital, Stockholm; Per Axelsson, Helsingborg Hospital, Helsingborg; Kjell Lundqvist, Örnsköldsvik Hospital, Örnsköldsvik; Gun Larsson, Blekinge Hospital, Karlshamn; Elena Holm, Malmö University Hospital, Malmö; Rose-Marie Kristoffersson, Mölndal Hospital, Mölndal; Cecilie Blimark, Sahlgrenska University Hospital, Göteborg; Maria Strandberg, Sundsvall Hospital, Sundsvall.

*Denmark*: Lene Meldgaard Knudsen and Per Trøllund Pedersen, Odense University Hospital, Odense; Peter Gimsing, Rigshospitalet, Köbenhavn; Niels Frost Andersen, Århus University Hospital, Århus; Annette Vangsted, Herlev Hospital, Köbenhavn; Henrik Gregersen, Aalborg University Hospital, Aalborg.

*Norway*: **Ø**yvind Hjertner, St Olavs Hospital, Trondheim; Nina Gulbrandsen, Ullevål University Hospital, Oslo; Fredrik Schjesvold, Diakonhjemmet, Oslo; Einar Haukås, Stavanger University Hospital, Stavanger.
